# Vena cava inferior thrombosis detected by venous hum: a case report

**DOI:** 10.1186/1752-1947-1-67

**Published:** 2007-08-22

**Authors:** Robert Colebunders, Britt Colebunders

**Affiliations:** 1 Department of clinical sciences, Institute of Tropical Medicine, Nationalestraat 155, 2000 Antwerp, Belgium; 2 Department of internal medicine, University of Antwerp, Universiteitsplein 1, 2610 Antwerp, Belgium; 3 Faculty of Medicine, University of Antwerp, Campus Drie Eiken, Universiteitsplein 1, 2610 Antwerp, Belgium

## Abstract

We describe a patient in which a venous hum, heard during abdominal auscultation, lead to the diagnosis of a vena cava inferior thrombosis.

## Background

An early diagnosis of a vena cava inferior thrombosis is essential because this is a life threatening condition if treatment is delayed. To diagnose a deep venous thrombosis (DVT) a clinical prediction score "the Wells scoring system" has been proposed (Table 1) [1]. However, even in patients with a DVT of the lower legs this scoring system was found to be of limited value and not able to replace the physicians empirical assessment [2,3]. Certainly for the diagnosis of a vena cava inferior thrombosis such a scoring system is not very useful. In the following case a venous hum, detected during abdominal auscultation, was almost the only clinical sign suggesting a vena cava inferior thrombosis.

**Table 1:** Wells Score for Clinical Risk Deep Vein Thrombosis

Active cancer (1 point)
Paralysis, paresis, or recent plaster immobilization of the lower extremity (1 point)
Recently bedridden for more than three days or major surgery within four weeks (1 point)
Localized tenderness along the distribution of the deep venous system (1 point)
Entire leg swollen (1 point)
Calf swelling by more than 3 cm when compared with the asymptomatic leg (1 point)
Pitting edema -greater in the symptomatic leg (1 point)
Collateral superficial veins-non-varicose (1 point)
Alternative diagnosis as likely or more possible than that of DVT (-2 points)

## Case presentation

A 24-year-old woman was admitted because of fever, headache, muscle pain and a mild erythematous rash after a trip to Costa Rica. She had been travelling by airplane for 12 hours. On physical examination a few small occipital lymph nodes were noted. Based on serological testing an acute toxoplasmosis infection was diagnosed. During 2 weeks of hospitalisation she nearly always stayed in bed because of extreme fatigue. She did not wear T.E.D. anti-embolism stockings and was not put on anticoagulation prevention. Since the age of 16, she took an oral anti-contraceptive (cyproteron-ethinylestradiol), but never smoked cigarettes. There was no family history of clotting disorder. After discharge she was not followed up actively.

Sixteen days after her first hospitalisation, she was readmitted because of diffuse abdominal pain and a slight degree of shortness of breath. On admission her blood pressure was 120/70 mmHg, pulse rate 90/min, temperature 36.3°C. The respiratory rate was not noted. There was no swelling of the legs. Examination of the lungs and the abdomen was normal except that abdominal auscultation revealed a murmur in the central area of the abdomen, slightly more to the right. Because of this finding, an urgent CT scan of the abdomen was performed. The CT scan revealed a thrombosis of the vena cava inferior, the left vena cava iliaca communis and externa and a small triangular pulmonary infiltrate at the lower part of the right lung. A chest X-ray and an electrocardiogram did not show any abnormalities. O^2 ^saturation was not measured. A ventilation perfusion isotopic scan confirmed the diagnosis of pulmonary embolism. A cavography showed a floating thrombus in the vena cava inferior. A temporary vena cava filter was installed and she was successfully treated initially with thrombolytic and later anticoagulant therapy. Investigations for genetic clotting disorders did not reveal any abnormality.

## Discussion

Auscultation of the abdomen of this patient, with a Wells score of only 1, lead to the diagnosis of a life threatening condition. This abdominal murmur was probably a venous hum caused by the floating thrombus in the vena cava inferior.

There are different types of venous hum. A cervical venous hum is a continuous noise heard over the internal jugular vein at the base of the neck [4,5]. It is frequently present in normal people [5]. A hepatic venous hum has been described in patients with liver cirrhosis, because of an arterio-venous shunt or constriction of the vena cava inferior by peri-venous hepatic fibrosis [6]. As far as we know, a venous hum due to vena cava thrombosis has not been reported.

Our patient presented with a floating thrombus in the vena cava inferior documented by cavography. A free-floating thrombus in the vena cava inferior is a high risk factor for pulmonary embolism. Radomski JS et al evaluated the risk of pulmonary embolism in 39 patients with phlebographically documented inferior vena cava thrombosis [7]. Twenty-six (67%) of them had thrombi characterized as free floating. The incidence of pulmonary embolism in those patients was 50% compared to 15% in those with adherent mural thrombi.

## Conclusion

A vena cava inferior thrombosis should be suspected in the presence of an abdominal venous hum. Today, with all the sophisticated technical diagnostic tools we have at our disposal, we sometimes forget that the old stethoscope still remains a very useful and cheap diagnostic instrument.

## Competing interests

The author(s) declare that they have no competing interests.

## Authors' contributions

RC was the doctor taking care of the patient; BC reviewed the literature; both RC and BC wrote the paper and approved the final manuscript.

## References

[B1] Wicki J Perneger TV Junod AF Bounameaux H Perrier A Assessing clinical probability of pulmonary embolism in the emergency ward: a simple score Arch Intern Med 2001 161 92 7 10.1001/archinte.161.1.92 11146703

[B2] Goodacre S Sutton AJ Sampson FC Meta-analysis: The value of clinical assessment in the diagnosis of deep venous thrombosis Ann Intern Med 143 2 129 39 2005 Jul 19 16027455 10.7326/0003-4819-143-2-200507190-00012

[B3] Douketis JD Use of a clinical prediction score in patients with suspected deep venous thrombosis: two steps forward, one step back? Ann Intern Med 143 2 140 2 2005 Jul 19 16027456 10.7326/0003-4819-143-2-200507190-00013

[B4] Groom D Boone JA Jenkins M Venous hum in cardiac auscultation J Am Med Assoc 159 7 639 41 1955 Oct 15 13263102 10.1001/jama.1955.02960240005002

[B5] Hardison JE Editorial: The cervical venous hum: a help and a hindrance N Engl J Med 292 23 1239 40 1975 Jun 5 1128577 10.1056/NEJM197506052922311

[B6] McFadzean AJ Gray J Hepatic venous hum in cirrhosis of liver Lancet 265 6796 1128 30 1953 Nov 28 13110079 10.1016/s0140-6736(53)91499-8

[B7] Radomski JS Jarrell BE Carabasi RA Yang SL Koolpe H Risk of pulmonary embolus with inferior vena cava thrombosis Am Surg 1987 53 2 97 101 3813221

